# Agreement in Left Ventricular Function Measured by Echocardiography and Cardiac Magnetic Resonance in Patients With Chronic Coronary Total Occlusion

**DOI:** 10.3389/fcvm.2021.675087

**Published:** 2021-06-15

**Authors:** Jiahui Li, Lijun Zhang, Yueli Wang, Huijuan Zuo, Rongchong Huang, Xueyao Yang, Ye Han, Yi He, Xiantao Song

**Affiliations:** ^1^Ward 1 of Coronary Heart Disease Center, Beijing Institute of Heart Lung and Blood Vessel Disease, Beijing Anzhen Hospital, Capital Medical University, Beijing, China; ^2^Department of Radiology, Beijing Institute of Heart Lung and Blood Vessel Disease, Beijing Anzhen Hospital, Capital Medical University, Beijing, China; ^3^Department of Echocardiology, Beijing Institute of Heart Lung and Blood Vessel Disease, Beijing Anzhen Hospital, Capital Medical University, Beijing, China; ^4^Department of Community Health Research, Beijing Institute of Heart Lung and Blood Vessel Disease, Beijing Anzhen Hospital, Capital Medical University, Beijing, China; ^5^Department of Cardiology, Beijing Friendship Hospital, Capital Medical University, Beijing, China

**Keywords:** magnetic resonance imaging, transthoracic echocardiography, left ventricular function, chronic total occlusion, agreement

## Abstract

**Aims:** To determine the agreement between two-dimensional transthoracic echocardiography (2DTTE) and cardiovascular magnetic resonance (CMR) in left ventricular (LV) function [including end-systolic volume (LVESV), end-diastolic volume (LVEDV), and ejection fraction (LVEF)] in chronic total occlusion (CTO) patients.

**Methods:** Eighty-eight CTO patients were enrolled in this study. All patients underwent 2DTTE and CMR within 1 week. The correlation and agreement of LVEF, LVESV, and LVEDV as measured by 2DTTE and CMR were assessed using Pearson correlation, Kappa analysis, and Bland–Altman method.

**Results:** The mean age of patients enrolled was 57 ± 10 years. There was a strong correlation (*r* = 0.71, 0.90, and 0.80, respectively, all *P* < 0.001) and a moderately strong agreement (Kappa = 0.62, *P* < 0.001) between the two modalities in measurement of LV function. The agreement in patients with EF ≧50% was better than in those with an EF <50%. CTO patients without echocardiographic wall motion abnormality (WMA) had stronger intermodality correlations (*r* = 0.84, 0.96, and 0.87, respectively) and smaller biases in LV function measurement.

**Conclusions:** The difference in measurement between 2DTTE and CMR should be noticed in CTO patients with EF <50% or abnormal ventricular motion. CMR should be considered in these conditions.

## Introduction

For patients with coronary chronic total occlusion (CTO), left ventricular (LV) function assessment before revascularization is crucial for clinical decision-making and has reference value in evaluating the improvement of cardiac function status after revascularization (1, 2). LV function can be measured by several non-invasive cardiac imaging modalities, including echocardiography, cardiac magnetic resonance (CMR), and cardiac computed tomography. Many studies regarding the comparison among these techniques have been reported. However, most of them enroll healthy subjects or patients with different cardiac diseases. Data about the agreement in ventricular function determined by these different modalities in CTO patients are still sparse.

The aim of this study was to determine the agreement between two-dimensional transthoracic echocardiography (2DTTE) and CMR in the assessment of LV function in CTO patients.

## Materials and Methods

### Study Population

**Figure 1:**
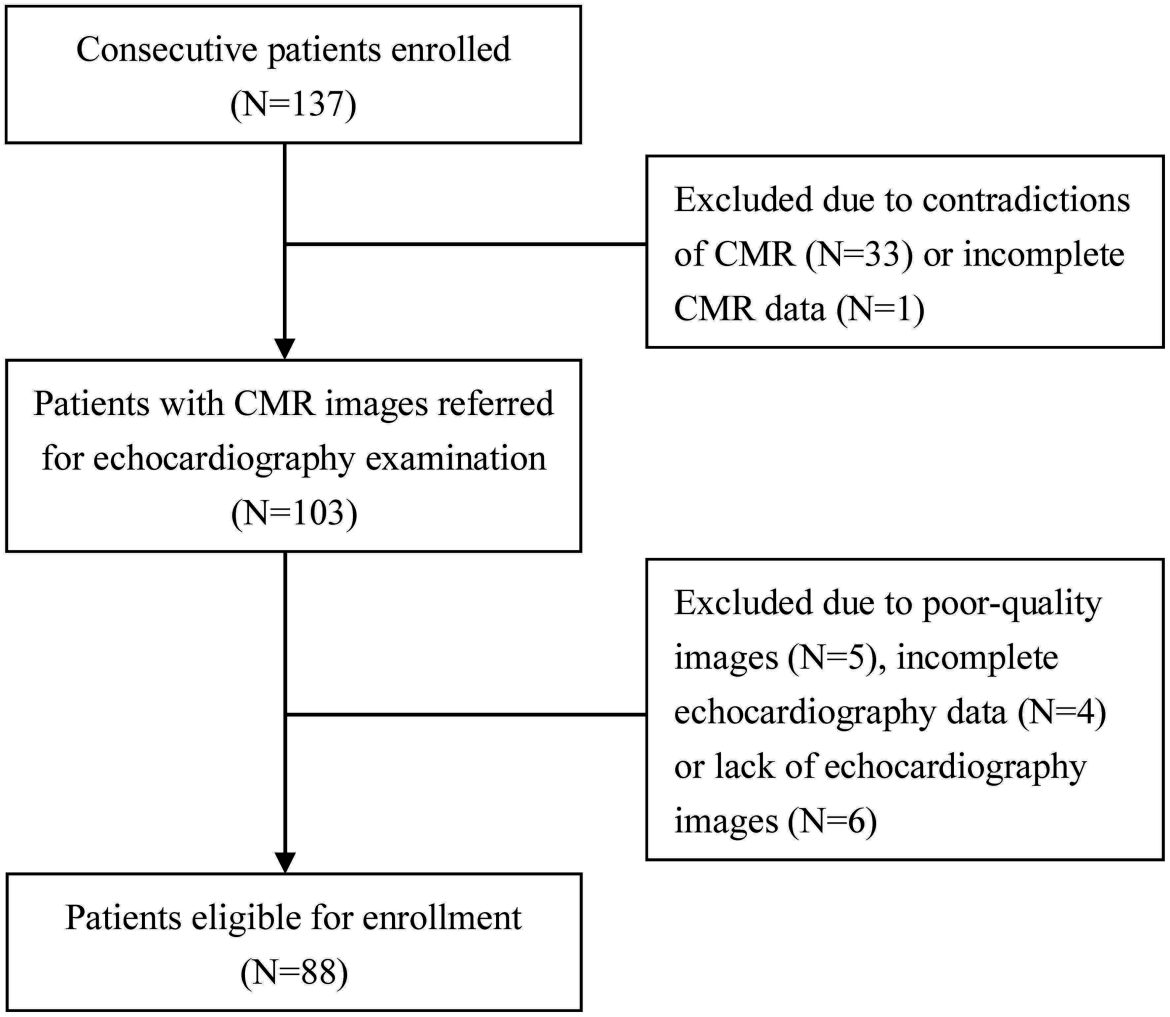
Flow chart of the study.

A total of 137 consecutive CTO patients, diagnosed by coronary angiography (CAG) from May 2015 to June 2017, were enrolled in this study. As shown in Figure 1, patients were excluded due to the contradictions of CMR (*N* = 33) or incomplete CMR data (*N* = 1). The rest of the patients were referred to echocardiography examination and further excluded due to poor-quality images (*N* = 5), incomplete 2DTTE data (*N* = 4), or lack of 2DTTE images (*N* = 6), leaving 88 patients eligible for the study. All enrolled patients underwent both echocardiography and CMR imaging within the same week. Coronary artery interventions were not performed until both types of imaging were finished. The coronary occlusion duration was divided into three levels: certain, likely, and undetermined, as previously described (3). The study was approved by Beijing Anzhen Hospital Ethics Committee, and the informed consent was waived.

### Transthoracic Echocardiography

Transthoracic echocardiography was assessed using standard clinical 2-dimensional imaging platforms: 56 patients with IE33 XMatrix (Philips, Amsterdam, Netherlands) and 32 patients with Vivid 7 (General Electric Medical Systems, Boston, USA). All images were recorded and analyzed by an experienced echocardiologist, who was blinded to clinical data and CMR results.

In each subject, assessments of LV function [including end-systolic volume (LVESV), end-diastolic volume (LVEDV), and ejection fraction (LVEF)] were performed according to the recommendations of the American Society of Echocardiography (4) following the Modified Simpson's rule (5). At end-diastole and end-systole stages, in apical four- and two-chamber views, the LV endocardial interface between myocardium and LV cavity was traced contiguously from one side of the mitral annulus to the other side, including papillary muscles as part of the LV cavity. The contour was finished after a straight line connected the two edges of mitral annular ring. LVEF, LVESV, and LVEDV were calculated using the biplane Simpson's formula.

For each patient, regional wall motion was assessed using a 17-segment LV model. The level of endocardial wall motion of each segment was scored according to previous guideline: score 1, normal or hyperkinetic; score 2, hypokinetic; score 3, akinetic; and score 4, dyskinetic (4). The wall motion score index (WMSI) was calculated by averaging the scores of 17 segments. Based on WMSI findings, two groups were identified: wall motion abnormality (WMA) group (WMSI = 1) and non-WMA group (WMSI > 1).

### Cardiac Magnetic Resonance Imaging

CMR imaging was performed using a 3-T whole-body scanner (Siemens, Munich, Germany) with a 32-element matrix coil. Images were obtained using steady-state and breath-hold cines in three long-axis planes and sequential short-axis slices extending from the mitral valve plane to just below the LV apex. End-diastolic and end-systolic volumes were obtained by manual delineation of the endocardial borders. Short-axis slices with ≧50% of the LV circumference surrounded by the myocardium were included in the process of volume calculation (6). The basal and apical slices were ensured on long-axis views. In every short-axis slice, the endocardial contour was traced at end-diastole and end-systole stages, with inclusion of papillary muscle and trabeculae as part of the LV cavity. Imaging analysis was performed by an experienced radiologist, who was blinded to the study. LVEF, LVESV, and LVEDV were calculated with commercially available software (CVI42 version5.9.1, Circle Cardiovascular Imaging, Calgary, AB, Canada). CMR was used as the reference standard for comparing with echocardiography.

### Statistical Analysis

All analyses were performed using SPSS (Version 20.0, IBM Corporation, Armonk, NY, USA). For continuous variables, a Shapiro–Wilk test was used for normal distribution tests. Normally distributed values were expressed as means ± standard deviation (SD) and compared using Student's *t*-test. Non-normally distributed ones were expressed as a median with interquartile range (IQR) and compared using the Mann–Whitney *U*-test. Categorical variables were expressed as percentages and compared using the chi-squared test. The intermodality correlation and agreement were tested using Pearson correlation, Kappa analysis, and Bland–Altman method, respectively. Bias and limits of agreement (LOA) were expressed as the mean and 95% confidence interval of the differences in normally distributed values and as median and 2.5th−97.5th percentiles of the differences in non-normally distributed values. All statistical tests were two-sided, and statistical significance was defined as *P* < 0.05.

## Results

### Patient Characteristics

A total of 88 patients (mean age, 57 ± 10 years; 83% male) with 90 CTO vessels were included in this study. Among them, 76 patients had presentation of angina (86.4%), 5 were diagnosed as non-ST-segment elevated myocardial infarction (NSTEMI) (5.7%), and 7 were asymptomatic (8.0%). Most of CTO lesions located in the right coronary artery (RCA) (44.4%), followed by left ascending branch (LAD) (40.0%) and left circumflex branch (LCX) (15.6%). The median interval between 2DTTE and CMR was 1 day. Further clinical data and LV functions are presented in Table 1.

**Table 1:** Baseline characteristics.

	**All patients *N =* 88**
Age (years)	57 ± 10
Male	73 (83.0)
**Clinical presentations**
Asymptomatic	7 (8.0)
Stable angina	8 (9.1)
Unstable angina	68 (77.3)
NSTEMI	5 (5.7)
**Duration of coronary occlusion**
Certain	45 (51.1)
Likely	10 (11.4)
Undetermined	33 (37.5)
Hypertension	49 (55.7)
Diabetes	18 (20.5)
Dyslipidemia	32 (36.4)
Prior myocardial infarction	24 (27.3)
Prior PCI	31 (35.2)
Smoking	47 (53.4)
Number of CTO vessels	90
**CTO location**
LAD	36 (40.0)
LCX	14 (15.6)
RCA	40 (44.4)
WMSI >1	40 (45.5)
Interval between 2DTTE and CMR (days)	1 (0–2)
**Echocardiography**
EF (%)	60.0 (54.3–63.9)
ESV (ml)	38.9 (32.0–53.8)
EDV (ml)	100.3 (82.5–123.9)
**CMR**
EF (%)	58.4 (50.3–66.5)
ESV (ml)	45.0 (28.8–56.8)
EDV (ml)	106.7 (83.3–130.3)

*Data are expressed as mean ± standard deviation or median with interquartile range*.

*CMR, cardiac magnetic resonance; CTO, chronic total occlusion; EF, ejection fraction; EDV, end-diastolic volume; ESV, end-systolic volume; NSTEMI, non-ST-segment elevated myocardial infarction; LAD, left ascending branch; LCX, left circumflex branch; PCI, percutaneous coronary intervention; RCA, right coronary artery; WMSI, wall motion score index; 2DTTE, two-dimensional transthoracic echocardiography*.

### Correlation and Agreement in 2DTTE and CMR Measurements of LV Function

**Figure 2:**
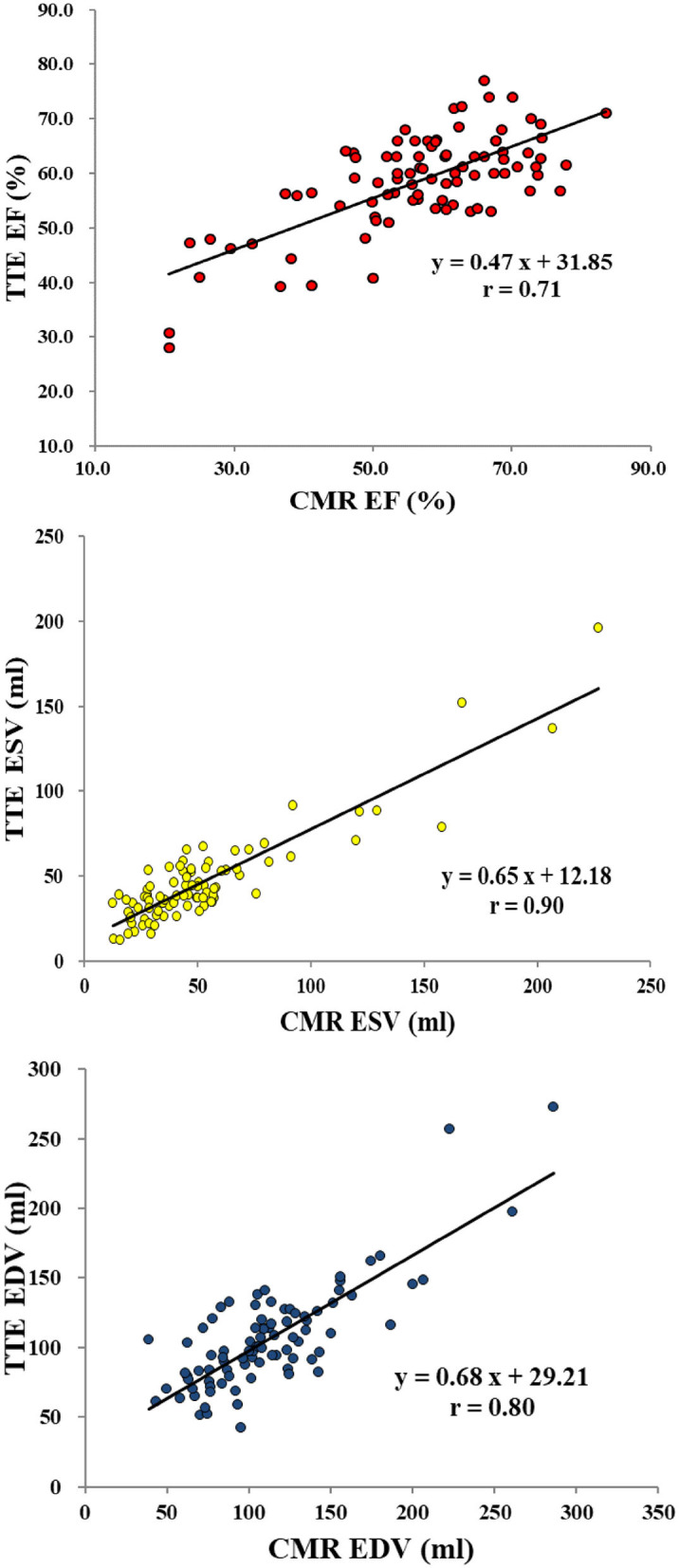
Linear regression analysis for left ventricular functions between echocardiography and CMR (reference standard). EF, ejection fraction; ESV, end-systolic volume; EDV, end-diastolic volume; CMR, cardiovascular magnetic resonance; TTE, transthoracic echocardiography.

**Figure 3:**
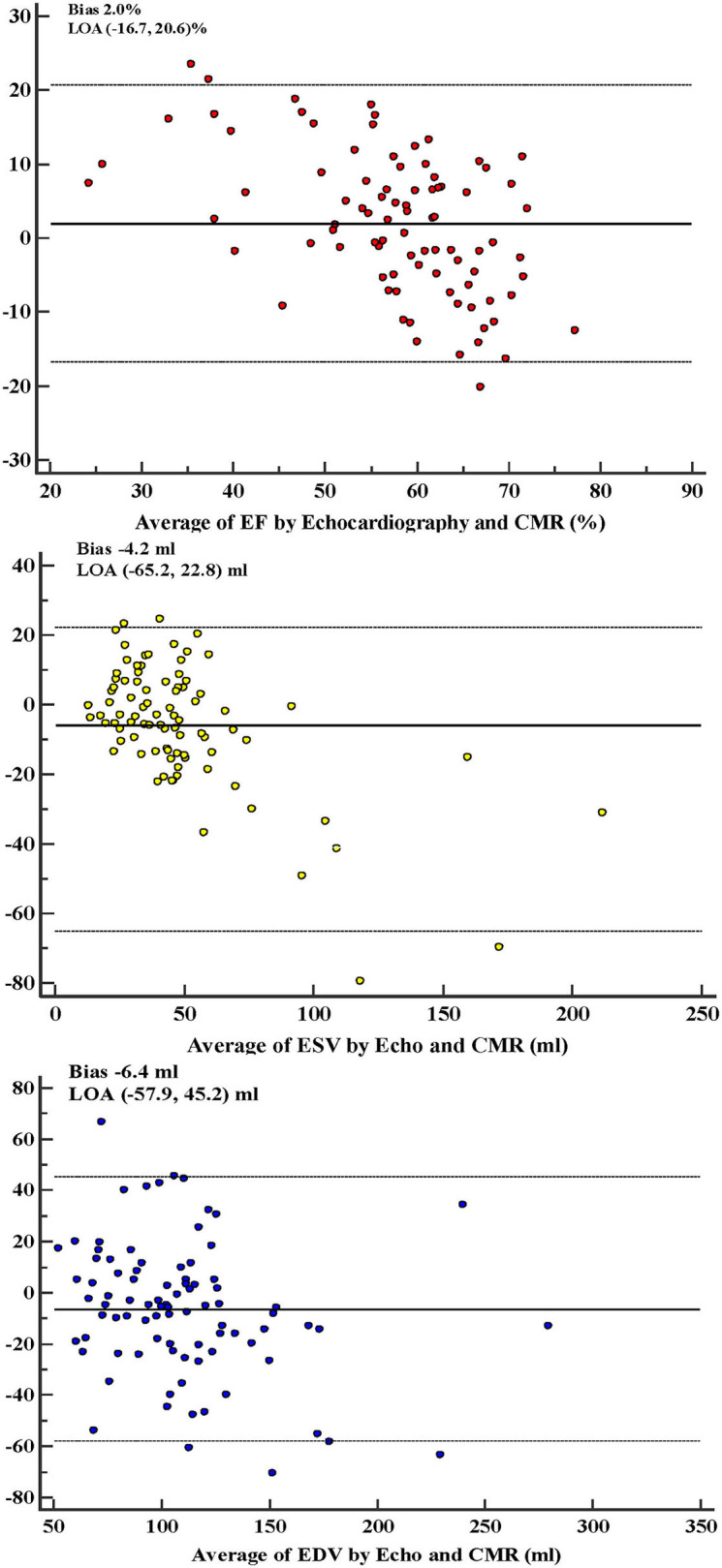
Bland–Altman analysis for left ventricular functions between echocardiography and CMR. Bias and 95% LOA were expressed as the mean and 95% confidence interval of the differences in normally distributed values (i.e., EF and EDV), as median and 2.5th−97.5th percentiles of the differences in non-normally distributed values (i.e., ESV). EF, ejection fraction; ESV, end-systolic volume; EDV, end-diastolic volume; CMR, cardiovascular magnetic resonance; LOA, limits of agreement.

In all 88 patients, correlation coefficients between 2DTTE and CMR for LVEF, LVESV, and LVEDV were 0.71, 0.90, and 0.80, respectively (*P* < 0.001 for all). (Figure 2, Table 2) The Bland–Altman analysis, bias, and 95% LOA between two modalities (with CMR as reference standard) were +2.0 (−16.7, 20.6)% for LVEF, −4.2 (−65.2, 22.8) ml for LVESV, and −6.4 (−57.9, 45.2) ml for LVEDV (Figure 3, Table 2). According to the heart failure guidelines (7), an LVEF of 50% was chosen as the threshold when assessing the agreement between 2DTTE and CMR. The intermodality agreement was moderately strong (*k* = 0.62, *P* < 0.001). In detail, 78 patients had the same classification when measured by 2DTTE and CMR, and 67 of them (85.9%) had an EF≧50%. 2DTTE reclassified 10 of the total 88 patients (11.4%). Furthermore, in 9 of the 10 instances (90.0%) of reclassification, 2DTTE-derived EF values were overestimated (≥50%) than CMR-derived EF (Table 3).

**Table 2:** Correlation and agreement analysis for 2DTTE and CMR.

	Pearson r^*^	**Linear regression**	**Bias^§^**	**limits of agreement^†^**
		**equation**		
EF	0.71	y = 0.47x + 31.85	2.0%	(−16.7, 20.6)%
ESV	0.90	y = 0.65x + 12.18	−4.2 ml	(−65.2, 22.8) ml
EDV	0.80	y = 0.68x + 29.21	−6.4 ml	(−57.9, 45.2) ml

*CMR, cardiac magnetic resonance; EF, ejection fraction; EDV, end-diastolic volume; ESV, end-systolic volume; 2DTTE, two-dimensional transthoracic echocardiography*.

* *All P values were < 0.001*.

§ *Bias in EF and EDV were expressed as the mean of the differences, as median of the differences in ESV*.

‡ *Limits of agreement in EF and EDV were expressed as the 95% confidence interval of the differences, as 2.5th−97.5th percentiles of the differences in ESV*.

**Table 3:** Patients assessed by echocardiography and CMR at EF 50% threshold.

		**CMR EF**		
		**≥50%**	**<50%**	**Total**
Echocardiographic EF	≥50%	67	9	76
	<50%	1	11	12
Total		68	20	88

*Kappa = 0.62, P < 0.001. CMR, cardiac magnetic resonance; EF, ejection fraction*.

### Agreement of Measurement for LV Functions in Patients With and Without Regional Wall Motion Abnormality

Based on 2DTEE findings, non-WMA group had 48 patients, and WMA group included 40 patients with 43 segments of abnormal wall motion. Most of segmental WMAs occur in the inferior wall of LV, and the distribution of WMA was in consistence with dominant area of occluded coronary arteries (Table 4). Among the five NSTEMI patients, only one had WMA in inferolateral wall of LV, rather than the dominant area of the CTO vessel (LAD).

**Table 4:** CTO vessels and the distribution of wall motion abnormalities.

	**LAD**	**LCX**	**RCA**	**Total**
Anterior	7	0	0	7
Anteroseptal	3	0	0	3
Anterolateral	4	3	0	7
Inferior	0	0	10	10
Inferoseptal	1	0	5	6
Inferolateral	1	2	7	10
Total	16	5	22	43

*CMR, cardiac magnetic resonance; CTO, chronic total occlusion; LAD, left ascending branch; LCX, left circumflex branch; RCA, right coronary artery*.

Compared with the WMA group, the non-WMA group had significantly higher CMR-derived EF (59.0 ± 10.4 vs. 54.9 ± 16.1%) (Table 5) and higher intermodality correlations for LVEF, LVESV, and LVEDV (0.84 vs. 0.66, 0.96 vs. 0.87, and 0.87 vs. 0.75, respectively). Additionally, bias in LVEF, LVESV, and LVEDV were all greater in WMA group (8.2 vs. 4.8%, −7.3 vs. −3.3 ml, and −12.3 vs. −10.6 ml, respectively) (Table 6).

**Table 5:** Comparison of patients with and without regional wall motion abnormality detected by 2DTTE.

	**WMA *N =* 40**	**Non-WMA *N =* 48**	***P*-value**
Age (years)	57 ± 10	56 ± 10	0.742
Male	33 (82.5)	40 (83.3)	0.918
**Clinical presentations**
Asymptomatic	4 (10.0)	3 (6.3)	0.801
Stable angina	4 (10.0)	4 (8.3)	1.000
Unstable angina	31 (77.5)	37 (77.1)	0.963
Myocardial infarction(NSTEMI)	1 (2.5)	4 (8.3)	0.475
Hypertension	25 (62.5)	24 (50.0)	0.240
Diabetes	7 (17.5)	11 (22.9)	0.530
Dyslipidemia	15 (37.5)	17 (35.4)	0.840
Prior myocardial infarction	11 (27.5)	13 (27.1)	0.965
Prior PCI	14 (35.0)	17 (35.4)	0.968
Smoking	20 (50.0)	27 (56.2)	0.588
Interval between 2DTTE and CMR	1 (0–2)	2 (0–2)	0.474
(days)			
Number of CTO vessels	41	49	
**Echocardiography**
EF (%)	58.7 ± 8.8	58.6 ± 9.3	0.901
ESV (ml)	38.7 (31.0–57.5)	39.0 (32.5–52.4)	0.782
EDV (ml)	109.1 ± 38.3	104.1 ± 37.0	0.890
**CMR**
EF (%)	54.9 ± 16.1	59.0 ± 10.4	0.007
ESV (ml)	43.8 (28.1–58.0)	45.3 (28.8–56.5)	0.802
EDV (ml)	105.6 (79.2–123.6)	108.5 (85.0–135.3)	0.379

*Data are expressed as mean ± standard deviation or median with interquartile range*.

*CMR, cardiac magnetic resonance; CTO, chronic total occlusion; EF, ejection fraction; EDV, end-diastolic volume; ESV, end-systolic volume; NSTEMI, non-ST-segment elevated myocardial infarction; 2DTTE, two-dimensional transthoracic echocardiography*.

**Table 6:** Correlation and agreement analysis for 2DTTE and CMR in patients with and without regional wall motion abnormality.

		Pearson r^*^	**Linear regression equation**	**Bias^§^**	**Limits of agreement^†^**
WMA	EF	0.66	y = 0.39x + 39.37	8.2%	(−20.1, 23.5)%
	ESV	0.87	y = 0.55x + 16.98	−7.3 ml	(−79.1, 24.5) ml
	EDV	0.75	y = 0.60x + 42.68	−12.3 ml	(−53.5, 28.9) ml
Without	EF	0.84	y = 0.75x + 14.08	4.8%	(−19.3, 28.8)%
WMA	ESV	0.96	y = 0.80x + 5.61	−3.3 ml	(−39.0, 16.7) ml
	EDV	0.87	y = 0.79x + 13.89	−10.6 ml	(−50.1, 29.0) ml

*CMR, cardiac magnetic resonance; EF, ejection fraction; EDV, end-diastolic volume; ESV, end-systolic volume; 2DTTE, two-dimensional transthoracic echocardiography; WMA, wall motion abnormality*.

* *All P values were < 0.001*.

§ *Bias in EF and EDV were expressed as the mean of the differences, as median of the differences in ESV*.

‡ *Limits of agreement in EF and EDV were expressed as the 95% confidence interval of the differences, as 2.5th−97.5th percentiles of the differences in ESV*.

## Discussion

In this study, the results suggested that (a) there were strong correlations between 2DTTE and CMR for LV volume measurement in CTO patients; (b) for CTO patients with LVEF <50%, the strength of intermodality agreement might be lower and the EF was overestimated by 2DTTE; and (c) CTO patients with WMA had lower intermodality correlation and greater bias in LV evaluation.

Both echocardiography and CMR are important non-invasive cardiac techniques for accurate and practical cardiac function assessment. CMR is considered as the gold standard for volumetric and EF assessment, with better tissue characterization and endocardium definition (7). Previous studies had shown a strong correlation and agreement between 2DTTE and CMR in LV measurement (8), which was further confirmed in this study. However, the strength of intermodality correlation for LVEF (*r* = 0.71) was found to be lower than that in prior studies (9, 10). The Bland–Altman analysis also showed a small bias in LV measurements but with a large range of agreement. These findings might result from worse ventricular functions in enrolled subjects. Due to long-term ischemic impairment, minimal infarction and restructure in local myocardium are more common in CTO patients, leading to abnormalities in cardiac structure and function (11) and affecting the myocardium mapping during measurement.

In a previous study investigating the agreement between 2DTTE and CMR, 44% of enrolled patients differed in LVEF classification (≦35, 35–50, >50%) when comparing the two modalities (12). Another study including patients with ST-elevation myocardial infarction showed low sensitivity (52%) and positive predictive values (54%) to detect LVEF <50% using 2DTTE (13). In our study, in patients with the same classification detected by 2DTTE and CMR, the majority had an EF ≧50%. On the other hand, most of the patients who were reclassified by 2DTTE had a CMR-derived EF <50%. This implied that the intermodality agreement might be better in patients with normal EF and the necessity of CMR for further accurate assessment of LV function in patients with EF <50%.

For patients in early stage of acute myocardial infarction, WMA can be observed due to temporary ventricular dysfunction. Among the five NSTEMI patients in this study, four of them had no transient or persistent WMA. Only one NSTEMI patient had WMA in inferolateral wall, which was more likely to be related to previous history of inferior myocardial infarction. Although the distribution of WMA was basically in consistence with occluded coronary arteries, no WMA was detected in the dominant area of CTO vessels in these five NSTEMI patients. The reason for the inconsistency was that timely revascularization avoids further ischemic impairment and ensured myocardial viability and normal ventricular motion.

Besides, no significant difference was found in the history of myocardial infarction in comparison of patients with and without WMA. This suggests that WMA is not appropriate for diagnosis of myocardial infarction existence. In patients with cardiac microcirculation dysfunction or with equivocal evidence of cardiac ischemia, they may present with tiny or unrecognized infarction and undetectable WMA in echocardiography. In this condition, late gadolinium enhancement (LGE) performed by CMR can be better in the recognition of infarction lesions.

In addition, lower strength of intermodality correlations for LV measurements and more significant bias in LVEF were observed in WMA group. The defect in the mechanism of echocardiography measurement was the main reason for this result. During the assessment and calculation of LV function, the interface between myocardium and ventricular cavity should be mapped at the end-diastole and end-systole stages in apical four and two-chamber views. The LV model is then made into a bullet shape and becomes the basis for measurements of volumetric values and EF calculation (4). A concise mapping may avoid potential error in model making and measurement. However, in patients with WMA, asymmetry of LV cavity and irregular myocardial motion add difficulties in mapping the ventricular interface and are therefore more likely to add inaccuracies in echocardiography measurements. Although echocardiography has lower cost and is more convenient in practice, for patients with WMA or even worse cardiac function, some clinical applications such as implantable cardiac defibrillator and cardiac supplement device will be restricted or overused due to the inaccurate assessments by echocardiography. Thus, for these patients, CMR should be considered for accurate and reliable ventricular evaluation.

Several limitations of this study need to be addressed. First, this was a single center study with a small number of patients. Second, only CTO patients who underwent 2DTTE and CMR were enrolled. It may be better to expand this study to newly emerging modalities, including 3D echocardiography and cardiac computed tomography. Previous studies have confirmed that 3D echocardiography is superior to the 2D method when it comes to LV function assessment (14), especially in patients with abnormal LV dilating motion or distortedly shaped LV.

## Data Availability Statement

The original contributions presented in the study are included in the article/supplementary material, further inquiries can be directed to the corresponding author/s.

## Ethics Statement

The studies involving human participants were reviewed and approved by Beijing Anzhen Hospital Ethics Committee. The patients/participants provided their written informed consent to participate in this study.

## Author Contributions

XY and YHa collected the patient data. JL, LZ, and YW analyzed data and were major contributors in writing the manuscript. HZ, RH, YHe, and XS took the revision of the manuscript. All authors agree to be accountable for the content of the work.

## Conflict of Interest

The authors declare that the research was conducted in the absence of any commercial or financial relationships that could be construed as a potential conflict of interest.
